# Multi-dimensional fusion: transformer and GANs-based multimodal audiovisual perception robot for musical performance art

**DOI:** 10.3389/fnbot.2023.1281944

**Published:** 2023-09-29

**Authors:** Shiyi Lu, Panpan Wang

**Affiliations:** ^1^School of Music and Dance, Shanxi Vocational University of Engineering Science and Technology, Jinzhong, China; ^2^School of Computer Information Engineering, Nanchang Institute of Technology, Nanchang, China

**Keywords:** multimodal robots, music performance art, audio-visual perception, fusion technology, Transformer models

## Abstract

**Introduction:**

In the context of evolving societal preferences for deeper emotional connections in art, this paper explores the emergence of multimodal robot music performance art. It investigates the fusion of music and motion in robot performances to enhance expressiveness and emotional impact. The study employs Transformer models to combine audio and video signals, enabling robots to better understand music's rhythm, melody, and emotional content. Generative Adversarial Networks (GANs) are utilized to create lifelike visual performances synchronized with music, bridging auditory and visual perception. Multimodal reinforcement learning is employed to achieve harmonious alignment between sound and motion.

**Methods:**

The study leverages Transformer models to process audio and video signals in robot performances. Generative Adversarial Networks are employed to generate visually appealing performances that align with the musical input. Multimodal reinforcement learning is used to synchronize robot actions with music. Diverse music styles and emotions are considered in the experiments. Performance evaluation metrics include accuracy, recall rate, and F1 score.

**Results:**

The proposed approach yields promising results across various music styles and emotional contexts. Performance smoothness scores exceed 94 points, demonstrating the fluidity of robot actions. An accuracy rate of 95% highlights the precision of the system in aligning robot actions with music. Notably, there is a substantial 33% enhancement in performance recall rate compared to baseline modules. The collective improvement in F1 score emphasizes the advantages of the proposed approach in the realm of robot music performance art.

**Discussion:**

The study's findings demonstrate the potential of multimodal robot music performance art in achieving heightened emotional impact. By combining audio and visual cues, robots can better interpret and respond to music, resulting in smoother and more precise performances. The substantial improvement in recall rate suggests that the proposed approach enhances the robots' ability to accurately mirror the emotional nuances of the music. These results signify the potential of this approach to transform the landscape of artistic expression through robotics, opening new avenues for emotionally resonant performances.

## 1. Introduction

Music, as a sublime human creation, possesses the remarkable ability to penetrate the depths of emotions, elicit resonance, and convey sentiments. In the wake of rapid technological advancements and the gradual maturation of robotic technologies, the realm of music is undergoing a synergistic convergence with technology, giving birth to the domain of multimodal robot music performance art. Through the fusion of audio-visual perception, robots cease to be mere imitators and instead emerge as creators, breathing new life and possibilities into the realm of musical art (Davies, 2000; Savage et al., 2021). This study embarks on an exploration of the field of multimodal robot music performance art that amalgamates auditory and visual perceptions, focusing on how robots can evolve into both creators and performers of music. It investigates how robots can forge emotional connections with human audiences, infusing fresh dynamism into the progression of this domain.

The genesis of robot music performance stems from the pursuit of amalgamating art with technology. The exploration within this domain transcends mere technological inquiry; it extends into an exploration of human creativity, emotional expression, and cultural representation. The synthesis of robotics and music enables us to transcend the confines of traditional music performance, unveiling novel avenues for creativity and expression, thereby infusing unprecedented freshness and innovation into the realm of musical artistry. Furthermore, robot music performances craft a new auditory experience for spectators, enveloping them in an ocean of music, inviting them to immerse, feel, and contemplate.

Within the nexus of art and technology's confluence, the realm of multimodal robot music performance is rapidly asserting itself. Numerous researchers have eagerly embarked upon endeavors to unravel the intricacies of seamlessly integrating robots and music (Wang et al., 2022). Nevertheless, amidst the intricate interplay of music and technology, a spectrum of challenges and questions persists.

Primarily, despite the capability of robots to convey emotions through multimodal fusion, achieving genuine emotional resonance remains a substantial challenge. While humans are profoundly influenced by emotions during music appreciation, enabling robots to precisely comprehend, express, and communicate emotions is an intricate task (Löffler et al., 2018). Furthermore, music performance art involves the expression of creativity and individuality. The challenge lies in endowing robots with unique styles and emotions while seamlessly incorporating creative elements within performances (Dimitrievska and Ackovska, 2020). Generated content by machines might lack the expressive versatility and dynamics of human performers. Additionally, achieving synchronization between robot actions and music during performances proves arduous. Even with the integration of rhythm and emotional expression, aligning robot movements with music, enhancing the performance's allure, necessitates solving intricate technical hurdles. Moreover, multimodal fusion necessitates algorithms and models from various domains, such as Transformers, Generative Adversarial Networks (GANs), and reinforcement learning. The integration of these diverse technologies to achieve high-quality robot music performances could entail intricate systems engineering and optimization. Lastly, in real-time music performances, interactivity, and immediacy are pivotal. Robots must adapt to audience reactions and environmental changes during live shows, imposing demands on the stability and swift responsiveness of systems.

Within the realm of multimodal robot music performance, several remarkable studies have yielded significant achievements. For instance, in the domain of robot instrument performance, some researchers have enabled robots to play diverse instruments, like string instruments and drums, by imitating human gestures and movements (Weinberg et al., 2020). Their methodologies integrate dynamics and motion planning, allowing robots to emulate the expressiveness and skill of human musicians. In the sphere of robot vocal performance, endeavors have been made to harness sound synthesis technology, enabling robots to sing using their own voices and mimic various singing styles (Torre et al., 2020). Profound explorations have taken place in emotional expression, sound synthesis, and lyric comprehension, imbuing robot music vocal performances with greater individuality. Simultaneously, in the direction of robot music composition, researchers have delved into utilizing deep learning techniques to autonomously generate music compositions. By analyzing extensive music datasets, they've trained robot music composition systems capable of creating compositions in different styles and emotions, ushering in novel possibilities for music creation (Baek and Taylor, 2020).

The motivation behind this study stems from both the insights gained from existing research and the desire to extend upon their findings. Despite some exploration in the realm of multimodal robot music performance, numerous unresolved issues persist. Confronting challenges related to emotional expression, creative communication, and the coordination of music and movement, we aspire to provide enhanced solutions to these problems through the methods proposed in this study.

The primary goal of this study is to achieve more expressive and emotionally resonant robot music performances by integrating audio-visual perception (Ghaleb et al., 2019). We used technologies such as Transformer model, GANs and multi-modal reinforcement learning to inject more artistic and creative elements into the robot music performance, covering music and dynamics.

Our research possesses distinct advantages. Firstly, we amalgamate various technologies in the realm of multimodal fusion, enabling robots to closely emulate the artistic aspects and emotional resonance of human music performances. Secondly, we emphasize the expression of creativity and individuality, enriching robot music performances with unique styles and artistic charm through the application of GANs. Lastly, we strive to achieve synchronization between music and movement. By employing multimodal reinforcement learning, we aim to make robot performances more captivating and enthralling.

In the field of multimodal robot music performance art, the intersection of technology and art opens up limitless avenues for exploration. Through this research, we aspire to inject new vigor and innovation into the music performance domain by fusing technology and art. We hope to provide audiences with richer and more immersive musical experiences. With unwavering dedication and innovation, we firmly believe that multimodal robot music performance art will continue to expand its unique domain, paving the way for a more splendid future in the realm of musical art.

The contributions of this paper can be summarized in the following three aspects:

1. In this study, we have introduced the Transformer model into the realm of multimodal robot music performance art, facilitating the fusion of audio and video signals. This integration has resulted in a seamless connection between music and movement. Leveraging the strengths of the Transformer model in sequence modeling and attention mechanisms, we enable robots to gain a deeper understanding of the rhythm, melody, and emotion of music. Through the application of the Transformer model, we have achieved synchronization between music and movement, culminating in harmonious and emotionally enriched music performances.

2. We have employed an innovative approach involving GANs, which has empowered robot music performances with a heightened sense of realism and captivation in the visual realm. By reflecting the emotions and emotional conveyance of music through visual performances, we have significantly amplified the artistic value and emotional expression of robot performances. Our method transcends the constraints of visual representation, enabling audiences to immerse themselves more profoundly in the emotional and affective dimensions of the music.

3. In this study, we introduce multi-modal reinforcement learning, enabling the robot to execute actions in real-time performances based on the emotions and rhythms of the music. By defining appropriate reward mechanisms, we enable the robot to continuously optimize its actions during the music performance, achieving coherence, and consistency with the music's dynamics and expression. This results in a more seamless and natural fusion of music and motion, creating an immersive artistic experience for the audience.

The logical structure of this paper is as follows: The second section provides an in-depth review of the relevant literature, comprehensively surveying the current landscape of multi-modal robot music performance by extensively examining existing research. This section analyzes the strengths and limitations of various methods, identifies unresolved issues, and lays the groundwork for guiding future research directions. The third section elaborates on the methods adopted in this study. It details the principles, network architectures, and implementation processes of the utilized algorithms, including the Transformer model, GANs, and multi-modal reinforcement learning. This comprehensive explanation ensures that readers gain a thorough understanding of the research methods employed. The fourth section encompasses the complete experimental procedures. It introduces the experimental environment, outlines data acquisition and preprocessing, defines evaluation metrics, showcases experimental results for different models and combination approaches, performs quantitative analysis, visualizes comparisons, and assesses the effectiveness of the proposed methods. The fifth section delves deeply into the discussion of research outcomes. It analyzes the significance of the results, summarizes the innovative aspects of the methods, reflects on limitations, and outlines prospective research directions. Lastly, the sixth section concludes the entire document, emphasizing the contributions, significance, and future prospects of the research work, providing readers with a concise overview of the core points of the paper.

## 2. Related work

In the field of robotic music performance art, the rapid advancement of artificial intelligence and robotics technology has sparked widespread attention and interest. By merging music, technology, and art, researchers are dedicated to creating robotic music performances that evoke emotional resonance, artistic expression, and multi-modal interaction (Sato and McKinney, 2022). This section will start by introducing the background and gradually lead into a comprehensive review of research progress relevant to our research questions. Ultimately, it will analyze the limitations of existing studies, clarifying the innovative aspects of our own research.

In today's society, music, as an art form that elicits emotional resonance and cultural heritage, has always enjoyed people's love and attention (Nijs and Nicolaou, 2021). With the continuous progress of technology, multi-modal robotic music performances are emerging as a novel avenue, providing people with more diverse and innovative musical experiences. Robotic music performance entails not only mastery of music fundamentals and skills but also the ability to convey emotions during musical rendition, creating resonance with audiences to achieve artistic expression.

Within the expansive realm of related research directions, researchers have successfully created a series of robot music performances that exhibit high artistic and emotional capabilities by integrating cutting-edge technological approaches. These accomplishments serve as invaluable inspiration and reference points for our study. For instance, in Qin et al. (2018), researchers harnessed the structure of music and emotions as driving forces to develop a dance system using humanoid robots. This system enabled robots to perform dances guided by the rhythm and emotions of the music (Cai et al., 2021). By effectively merging the structural and emotional components of music, the robot's dance performances showed remarkable progress in terms of style diversity and behavioral novelty, showcasing the potential of robots in the realm of music and dance. On another front, Li et al. (2020) delved into the fusion of multi-modal information for the automated assessment of the aesthetic value of robot dance poses. Researchers combined visual and non-visual data and employed machine learning techniques to automatically evaluate the aesthetic quality of robot dance poses. This study not only provided an automated aesthetic assessment approach for robot dance creation but also demonstrated innovative technological applications within the realm of art. Additionally, researchers have explored the integration of music education and robot technology. In Shahab et al. (2022), the combination of virtual reality technology and robots was utilized to offer music education to children with autism. Through virtual music education programs, children were able to engage in music learning within a simulated environment, enhancing their social and cognitive skills. This research explored the potential of robot technology in music education for special populations, ushering in new possibilities within the field of music education. Lastly, Cosentino and Takanishi (2021) emphasized the significance of music as an art form and a mode of communication, as well as the crucial role of interaction between artists and their environment during the artistic process. The authors highlighted that artists need to excel not only in artistic skills but also in effectively interacting with audiences and fellow performers on various communication levels. In the domain of music performance, these interactions are often conveyed through subtle auxiliary gestures to avoid auditory disruptions (Ning et al., 2023). The outcomes of this literature provide us with profound reflections on artistic expression and emotional conveyance. Simultaneously, it serves as a testament to how technology can reach levels comparable to human performers within the realm of music performance.

However, despite the remarkable achievements made in the field of robot music performance, there still exist several limitations and unresolved issues in existing research (Ran et al., 2023). Firstly, current robot music performances face challenges in the realm of multi-modal fusion. While some studies attempt to integrate multi-modal information such as music, motion, and emotions, maintaining coherence and smoothness in this fusion while achieving harmonious synchronization remains a challenge. Moreover, emotional conveyance and artistic expression are also quandaries within the domain of robot music performance. Although some research endeavors have achieved emotional conveyance through music emotion analysis and motion design, how to infuse greater artistic expression and emotional resonance into robot performances remains an unsolved puzzle. Concurrently, in the amalgamation of music and technology, the cohesiveness and consistency between robot actions and music continue to be problematic. While existing studies have explored guiding robot actions using music rhythm and emotions, refining the alignment between highly coordinated music and motion remains an aspect requiring further enhancement. This limitation restricts the expressive potential and emotional conveyance of robot music performances.

To surmount the limitations and issues inherent in prior research, our study employs an array of advanced technological approaches to realize robot music performances that are more expressive and emotionally resonant. Foremost, we introduce the Transformer model, capitalizing on its prowess in sequence modeling and attention mechanisms (Wang et al., 2021). By amalgamating audio and video signals, we establish a tight connection between music and motion, enabling robots to better comprehend the rhythm, melody, and emotion of the music. Subsequently, we creatively incorporate GANs to translate the emotional and emotional conveyance aspects of the music into visual performances, elevating the artistic and emotional expression capacities of robot performances. Moreover, we introduce multi-modal reinforcement learning, allowing robots to execute corresponding actions based on the emotional quality and rhythm of the music, achieving harmonious synchronization between music and motion. By defining appropriate reward mechanisms, we continuously optimize robot actions throughout the music performance, ensuring coordination and congruence with the music, thereby delivering a more immersive artistic experience to the audience. In summary, the innovation of this study lies in the integration of advanced technologies such as the Transformer model, GANs, and multi-modal reinforcement learning, addressing the deficiencies of prior research and achieving better coordination between music and robot actions, resulting in music performances that are more artistically expressive and emotionally evocative.

To conclude, this paper thoroughly reviews and synthesizes literature from various domains related to robot music performance art, highlighting the strengths, limitations, and outstanding issues within existing research. By referencing representative studies, we delve into the current state of development in the field of multi-modal robot music performance art, spotlighting key challenges and potential opportunities. Our innovative approach, through the incorporation of various advanced technical methods, deepens our understanding of the problem and proposes solutions. Our innovative methods seamlessly fuse music, emotion, and motion, realizing a higher level of musical performance art. Looking ahead, we anticipate that our study will provide new perspectives for in-depth research in multi-domain fusion, emotional conveyance, and artistic expression, opening up new possibilities for the convergence of robot technology and the arts.

## 3. Methodology

**Figure 1:**
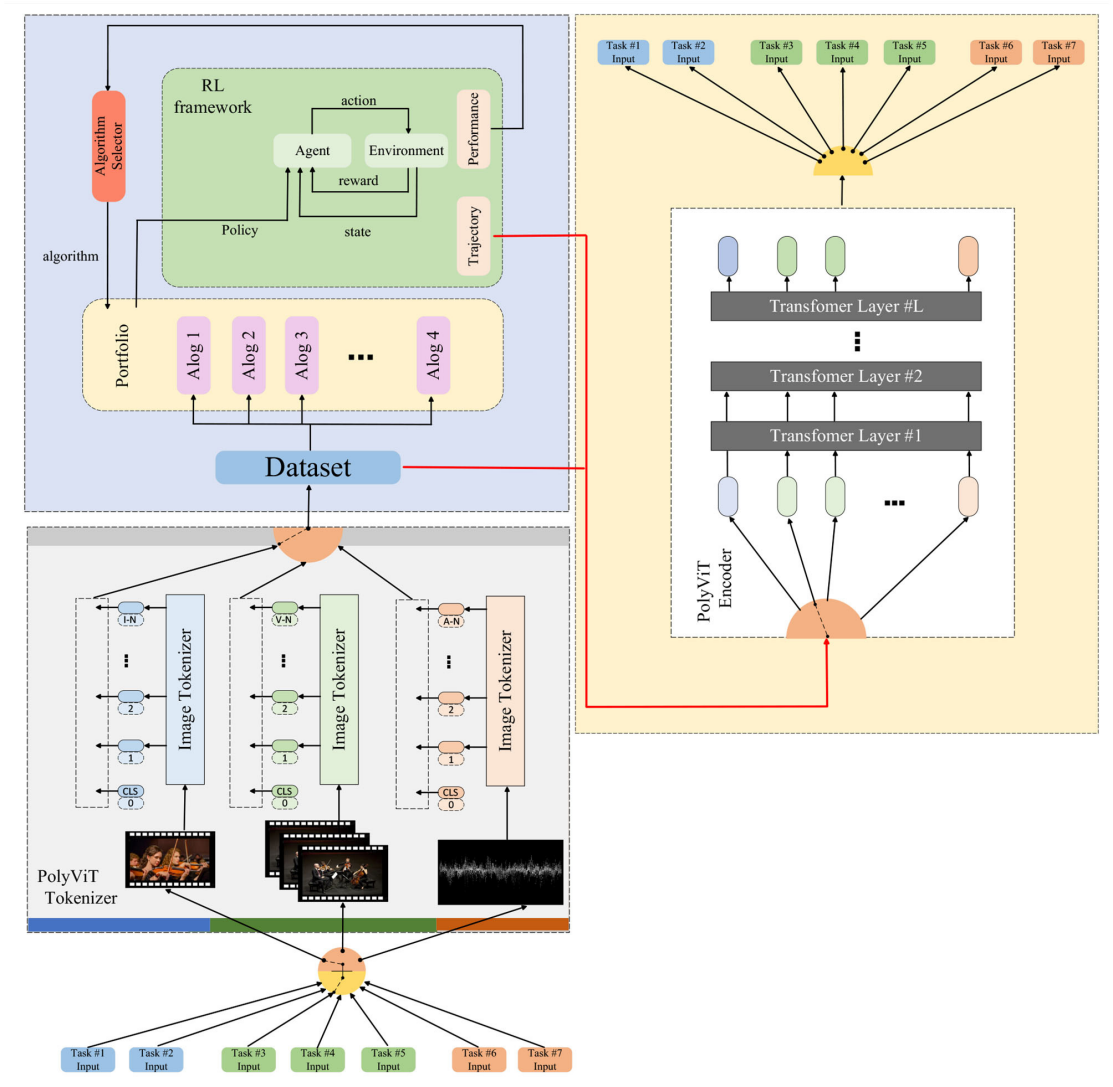
Overall algorithm flowchart.

In this study, due to the good performance of transformers in the field of music generation (Huang et al., 2023; Wang et al., 2023), we decided to introduce the transformer model into the field of multimodal robotic music performance art. We will provide a complex exposition of the comprehensive algorithms used to achieve expressive and emotionally resonant robotic musical performances. Through the fusion of a series of cutting-edge technical methods, we promote the seamless integration of music, emotion, and movement, thereby taking the art of music performance to a higher level. In order to clearly demonstrate our method, the overall algorithm flow chart is shown below Figure 1.

### 3.1. Transformer model

**Figure 2:**
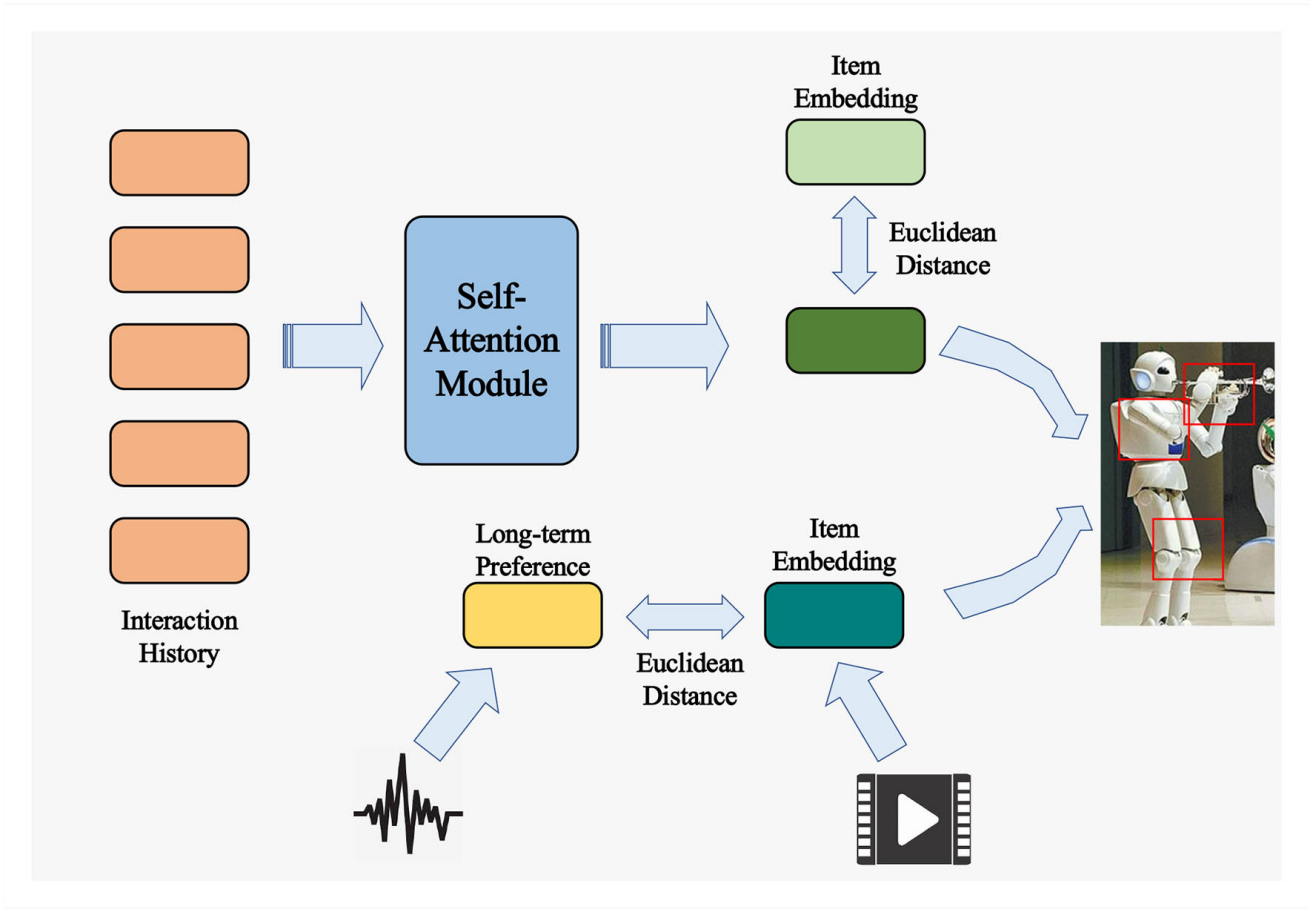
Transformer model.

When addressing the pursuit of more expressive and emotionally resonant robot music performances, the Transformer model emerges as a potent tool for sequence modeling, showcasing remarkable potential. The Transformer model, founded upon an attention-based neural network architecture (Gao et al., 2022), was initially devised for the realm of natural language processing. However, its exceptional performance in sequence modeling has engendered its widespread application across diverse domains. The framework of the Transformer model is illustrated in Figure 2 below.

In the Transformer model, the most essential component is the self-attention mechanism, which allows the model to assign varying attention weights to different positions of the input sequence, enabling it to capture contextual relationships within the sequence (Zhu et al., 2019). By calculating attention scores between each position and every other position, the Transformer model can capture the context in the input sequence.

The self-attention mechanism is a method for computing the correlation between any two elements in a sequence. Given a sequence *X* = (*x*_1_, *x*_2_, …, *x*_*n*_), where *x*_*i*_ represents the i-th element, we first map it into three distinct vectors: a query vector *q*_*i*_, a key vector *k*_*i*_, and a value vector *v*_*i*_. Subsequently, we compute the dot product between each query vector and all key vectors, scaled by a factor *d*_*k*_ (representing the dimension of key vectors). Next, a softmax operation is applied row-wise to obtain attention weights *a*_*ij*_ for each position. Finally, the output *z*_*i*_ is obtained by element-wise multiplication of each value vector with its corresponding attention weight, followed by summation. The formula is as follows:


(1) $$\begin{array}{lll} q_{i} & = & W_{q}x_{i}k_{i}=W_{k}x_{i}v_{i}=W_{i}x_{i}a_{ij} \end{array}$$

(2) $$q_{i}=\frac{\exp(q_{i}\cdot k_{j}/\sqrt{d_{k}})}{\sum_{j=1}^{n}\exp(q_{i}\cdot k_{j}/\sqrt{d_{k}})}z_{i}$$

(3) $$\begin{array}{lll} q_{i} & = & \overset{n}{\underset{j=1}{\sum }}a_{ij}v_{j} \end{array}$$

*W*_*q*_, *W*_*k*_, and *W*_*v*_ are linear transformation matrices representing the transformations for queries, keys, and values, respectively. They map the input sequence X into different representation spaces, enhancing the model's expressive power. *q*_*i*_, *k*_*i*_, and *v*_*i*_ denote the query, key, and value vectors of the i-th element, respectively. They are obtained through linear transformations applied to the input sequence X. *d*_*k*_ represents the dimension of the key vectors, serving as a constant factor to scale the dot product result, preventing numerical instability. *a*_*ij*_ represents the attention weight from the i-th position to the j-th position. It is a scalar value indicating the correlation between two positions. *z*_*i*_ represents the output vector of the i-th position, a vector that aggregates information from all positions.

In our research, we applied the Transformer model to model the relationship between music and motion. We utilized audio and video signals as input sequences, encoding them separately using the Transformer model. By fusing the encoded audio and video information, we achieved a close association between music and motion. Specifically, during the fusion stage, we employed another attention mechanism to compute the attention scores between the encoded audio and video representations, facilitating the alignment of music and motion. The calculation process is as follows:


(4) $$\begin{array}{l} \text{Attention}\left(A,V\right)=\text{softmax}\left(\frac{AV^{T}}{\sqrt{d_{k}}}\right)A \end{array}$$

Here, *A* represents the audio encoding and *V* represents the video encoding. Through this approach, we are able to establish effective connections between data from different modalities, thereby achieving coherence between music and motion.

To further optimize our model, we introduce the optimization function of the Transformer model, which is a variant of the Adam algorithm (Jais et al., 2019). This variant incorporates learning rate warm-up (Shazeer and Stern, 2018) and decay (Loshchilov and Hutter, 2018) strategies. Learning rate warm-up involves gradually increasing the learning rate at the beginning of training to prevent premature convergence to local optima. Learning rate decay involves gradually decreasing the learning rate toward the end of training to stabilize convergence. The formula is as follows:


(5) $$\begin{array}{l} \;lrate=\\ d_{model}^{-0.5}\cdot \min(step\_num^{-0.5},step\_num\cdot warmup\_steps^{-1.5}) \end{array}$$

Here, *d*_*model*_ represents the dimensionality of the model, *step*_*num*_ denotes the current training step, and *warmup*_*steps*_ represents the number of warm-up steps.

By training and fine-tuning our Transformer model on a large-scale dataset of music and motion, we enable the model to better capture the associations between music and motion, providing a robust foundation for sequence modeling in robot music performance. In the next section, we will elaborate on how we enhance the artistic and emotional expression of robot performance through the use of GANs.

### 3.2. Generative adversarial networks

**Figure 3:**
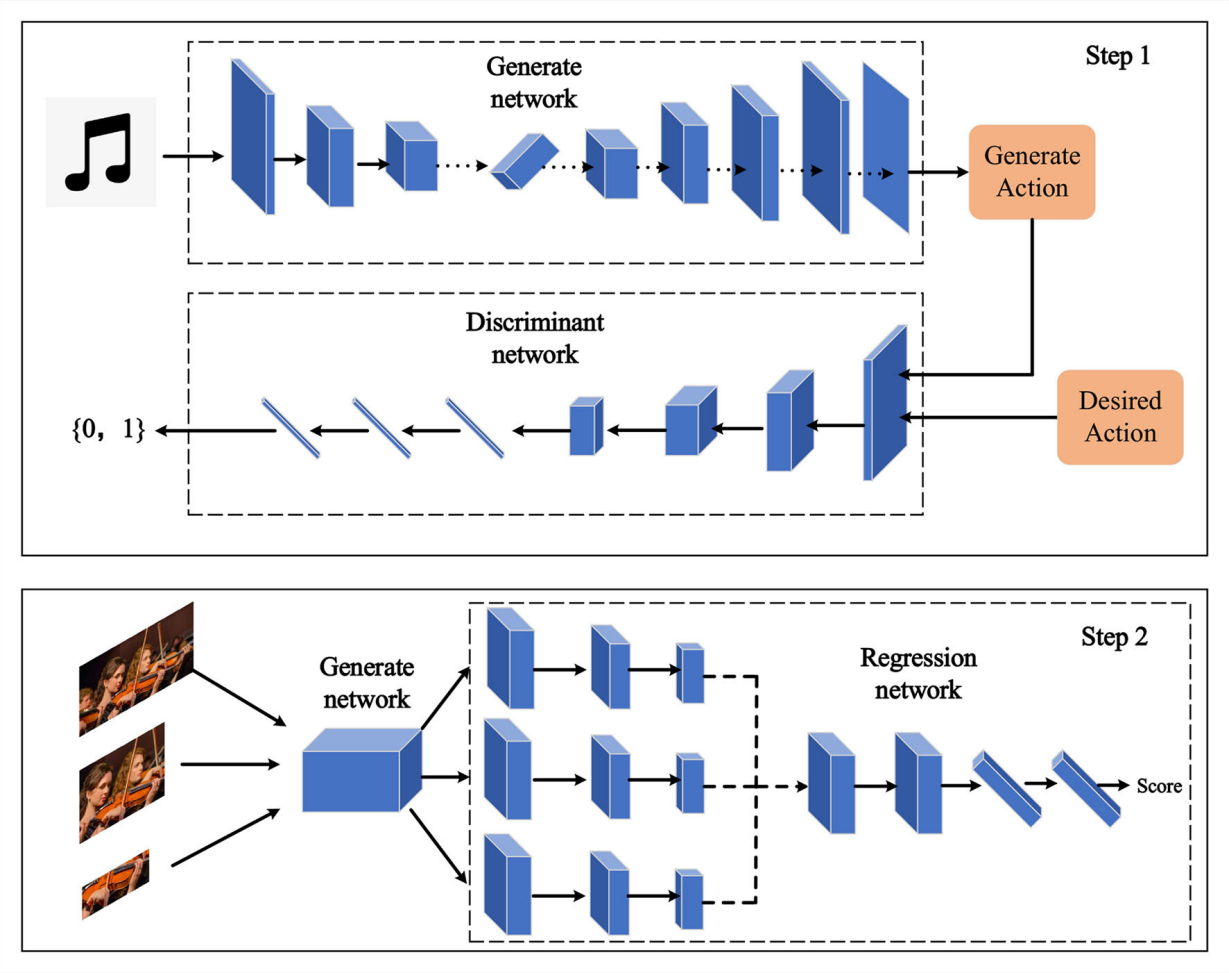
Generative adversarial network.

To further elevate the artistic quality and emotional expression of robot performance, we introduce GANs, a potent deep learning architecture widely employed for generating lifelike data (Jin et al., 2019; Aggarwal et al., 2021). The structure of a Generative Adversarial Network model is depicted in Figure 3 below.

A Generative Adversarial Network comprises two components: the generator and the discriminator (Lu et al., 2022). The generator aims to produce realistic data samples, while the discriminator aims to differentiate between samples generated by the generator and real samples. These two components engage in a competitive process through adversarial training, causing the generator to progressively generate more realistic data samples. The update process for the generator in a Generative Adversarial Network can be expressed through the following formula:


(6) $$\begin{array}{l} \;\min_{G}\max_{D}V(D,G)=\\ {𝔼}_{x\sim p_{\text{data }}(x)}[\log D(x)]+{𝔼}_{z\sim p_{z}(z)}[\log(1-D(G(z)))] \end{array}$$

Where *G* represents the generator, *D* represents the discriminator, *x* stands for real samples, *z* represents random noise, *p*_*data*_(*x*) denotes the distribution of real samples, and *p*_*z*_(*z*) represents the noise distribution. The objective of the generator is to minimize the probability of the discriminator's error, leading to the generation of realistic samples. The update process for the discriminator can be outlined as follows:


(7) $$\begin{array}{l} \max_{D}V(D,G)={𝔼}_{x\sim \rho_{\text{data}}(x)}[\log D(x)]\\ \;+{𝔼}_{z\sim p_{z}(z)}[\log(1-D(G(z)))] \end{array}$$

The discriminator's objective is to maximize the probability of correctly distinguishing between real and generated samples. By iteratively training the generator and the discriminator, the generative adversarial network gradually reaches an equilibrium where the realism of the generated samples improves over time.

In our research, we apply the generative adversarial network to enhance the emotional expression of robot performances. Specifically, our generator takes music information as input and generates visual performances that match the emotional content of the music. The discriminator evaluates whether the generated performance is consistent with real samples. Through this approach, we enable the robot's performances to convey emotions more effectively, thereby enhancing their artistic quality.

To optimize our generative adversarial network (GANs), we introduce the optimization function of GANs, which is an algorithm based on stochastic gradient descent (SGD) (Newton et al., 2018) or its variants (such as Adam). This algorithm updates the network's parameters according to the gradient of the loss function. Specifically, there are several formulas as follows:


(8) $$\begin{array}{l} \theta_{D}\leftarrow \theta_{D}-\alpha \nabla_{\theta_{D}}L_{D}\theta_{G}\leftarrow \theta_{G}-\alpha \nabla_{\theta_{G}}L_{G} \end{array}$$

Where *θ*_*D*_ and *θ*_*G*_ represent the parameters of the discriminator *D* and the generator *G*, α denotes the learning rate, and ∇ indicates the gradient operator.

In the next section, we will elaborate on how to achieve the coordination between music and motion through reinforcement learning, thereby further enhancing the quality of robot music performance.

### 3.3. Reinforcement learning

To achieve coordination between music and robot motion, we introduce reinforcement learning, a machine learning approach used to train agents to achieve target tasks by learning optimal strategies through continuous interaction with the environment (Sutton and Barto, 2018).

**Figure 4:**
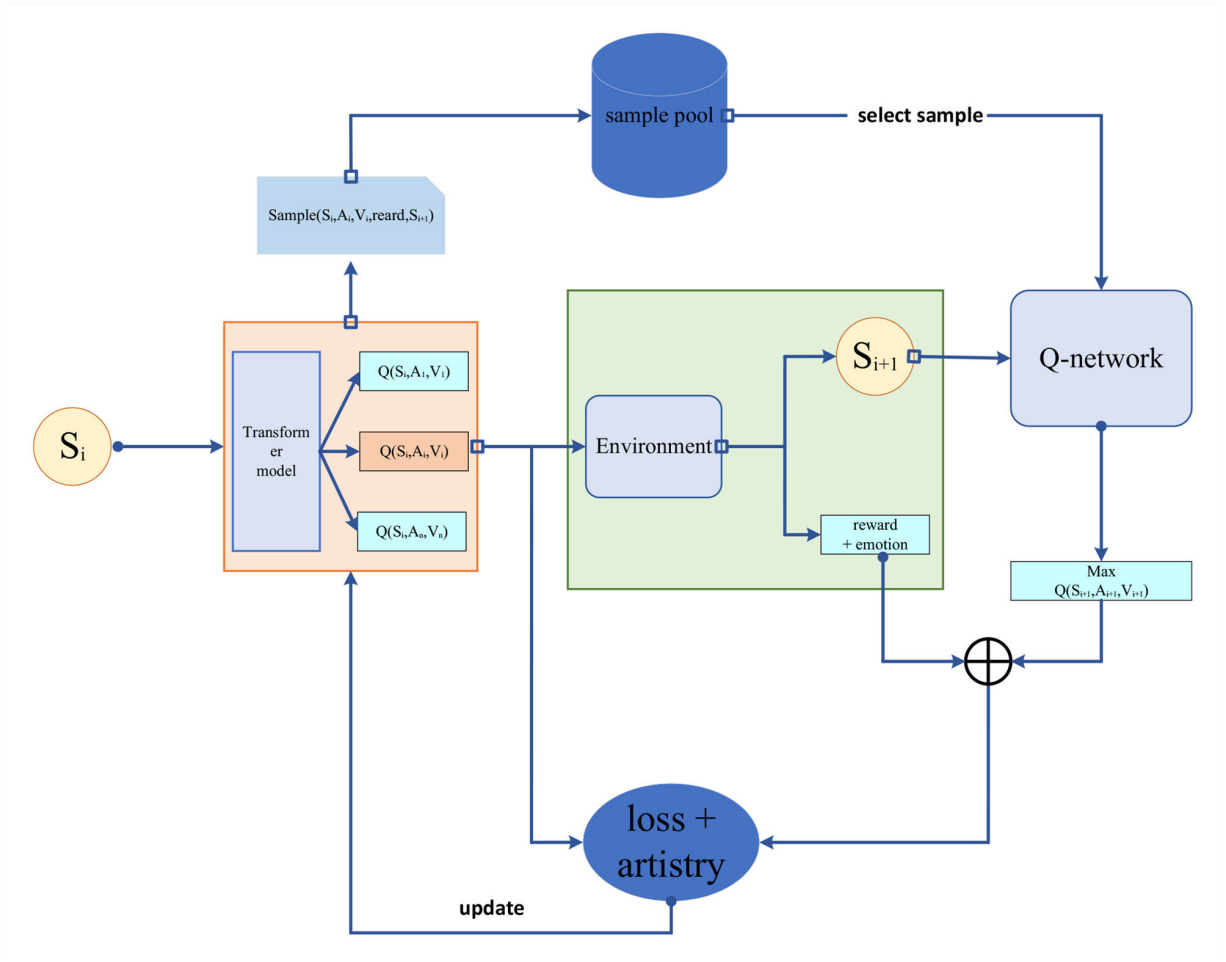
Reinforcement learning.

Key concepts in reinforcement learning include states, actions, rewards, and policies. An agent selects actions based on the current state, interacts with the environment, and receives rewards, gradually optimizing its policy to maximize cumulative rewards. The reinforcement learning model is depicted in Figure 4 below.

The process of reinforcement learning can be described using a Markov Decision Process (MDP) (Altman, 2021). In an MDP, the state space *S* describes the possible states of the agent, the action space *A* describes the possible actions of the agent, the transition probability function P(s′∣s, a) describes the probability of transitioning to state *s*′ after taking action *a* in state *s*, and the reward function R(s, a, s′) represents the reward obtained when transitioning from state *s* to state *s*′ by taking action *a*. The agent's policy can be represented as π (a∣s), indicating the probability of selecting action *a* in state *s*.

The goal of reinforcement learning is to find an optimal policy π^*^, which allows the agent to achieve the maximum cumulative reward when following that policy. The cumulative reward can be defined as the sum of all reward values obtained from the current state to the terminal state or can incorporate a discount factor γ to consider the impact of future rewards on current decisions. The formula is as follows:


(9) $$\begin{array}{l} G_{t}=R_{t+1}+R_{t+2}+R_{t+3}+...+R_{T}G_{t}=R_{t+1}+\gamma R_{t+2}\\ \;+\gamma^{2}R_{t+3}+...=\sum_{k=0}^{\infty }\gamma^{k}R_{t+k+1} \end{array}$$

Where *G*_*t*_ represents the cumulative reward starting from time step *t*, *R*_*t*_ represents the immediate reward at time step t, T represents the terminal time step, and γ is the discount factor, which is a constant between 0 and 1.

To evaluate and compare the effectiveness of different policies, several metrics are commonly used: state-value function, action-value function, optimal state-value function, and optimal action-value function. There are relationships between these functions, such as:

- The state value function *V*_π_(*s*) signifies the anticipated cumulative reward obtainable by following a given policy π in a specific state *s*. It is equivalent to the weighted sum of the probabilities of selecting different actions in that state, multiplied by their corresponding action value function *Q*_π_(*s, a*). The formula is as follows:
  (10) $$\begin{array}{l} V_{\pi }\left(s\right)=\underset{a\in A}{\sum }\pi \left(a|s\right)Q_{\pi }\left(s,a\right) \end{array}$$
- The action value function *Q*_π_(*s, a*) represents the anticipated cumulative reward achievable by taking a specific action *a* in a particular state *s* and then adhering to a given policy π. It is the weighted sum of the immediate reward obtained by taking that action in the current state, denoted as *R*(*s, a*), and the state value function $V_{\pi }\left(s^{\prime }\right)$ of the subsequent state *s*′ resulting from following policy π after the transition. The transition probability is represented as *P*(*s*′|*s, a*) and the discount factor is denoted as γ. The formula is as follows:
  (11) $$Q_{\pi }(s,a)=R(s,a)+\gamma \sum_{s^{'}\in S}P(s^{'}|s,a)V_{\pi }(s^{'})$$
- The optimal state value function V^*^(*s*) signifies the maximum expected cumulative reward achievable by adhering to the optimal policy π^*^ when starting from a specific state *s*. It is equivalent to selecting the maximum optimal action value function Q^*^(*s, a*) among all possible actions in that state. The formula is as follows:
  (12) $$\begin{array}{l} V^{*}\left(s\right)=\underset{a\in A}{\max}Q^{*}\left(s,a\right) \end{array}$$
- The optimal action value function Q^*^(*s, a*) signifies the maximum expected cumulative reward achievable by taking a specific action *a* in a certain state *s* and following the optimal policy π^*^. It is equivalent to the weighted sum of the immediate reward *R*(*s, a*) obtained by taking the action and the optimal state value function V^*^(*s*′) of the next state *s*′ when following the optimal policy π^*^. The weighting considers the transition probability *P*(*s*′|*s, a*) and is influenced by the discount factor γ. The formula is as follows:
  (13) $$Q^{*}(s,a)=R(s,a)+\gamma \sum_{s^{'}\in S}P(s^{'}|s,a)V^{*}(s^{'})$$

Our reinforcement learning approach aims to enable the robot to select appropriate actions based on the emotions and rhythm of the music, achieving coordination between music and actions. We employ deep reinforcement learning techniques, training neural networks to approximate the state value function and action value function. Our optimization objective is to maximize the cumulative reward, where the reward function is closely tied to the emotions and rhythm of the music to ensure consistency between the robot's performance and the music.

In the next chapter, we will provide a detailed description of the experimental setup we designed, along with the results obtained from the experiments. Through practical data and analysis, we will validate the effectiveness and performance of our method. By showcasing the results of our experiments, we will further solidify the standing of our approach in the realm of multimodal robot music performance art. Moreover, these results will serve as a robust reference for future research and development endeavors.

## 4. Experiment

**Figure 5:**
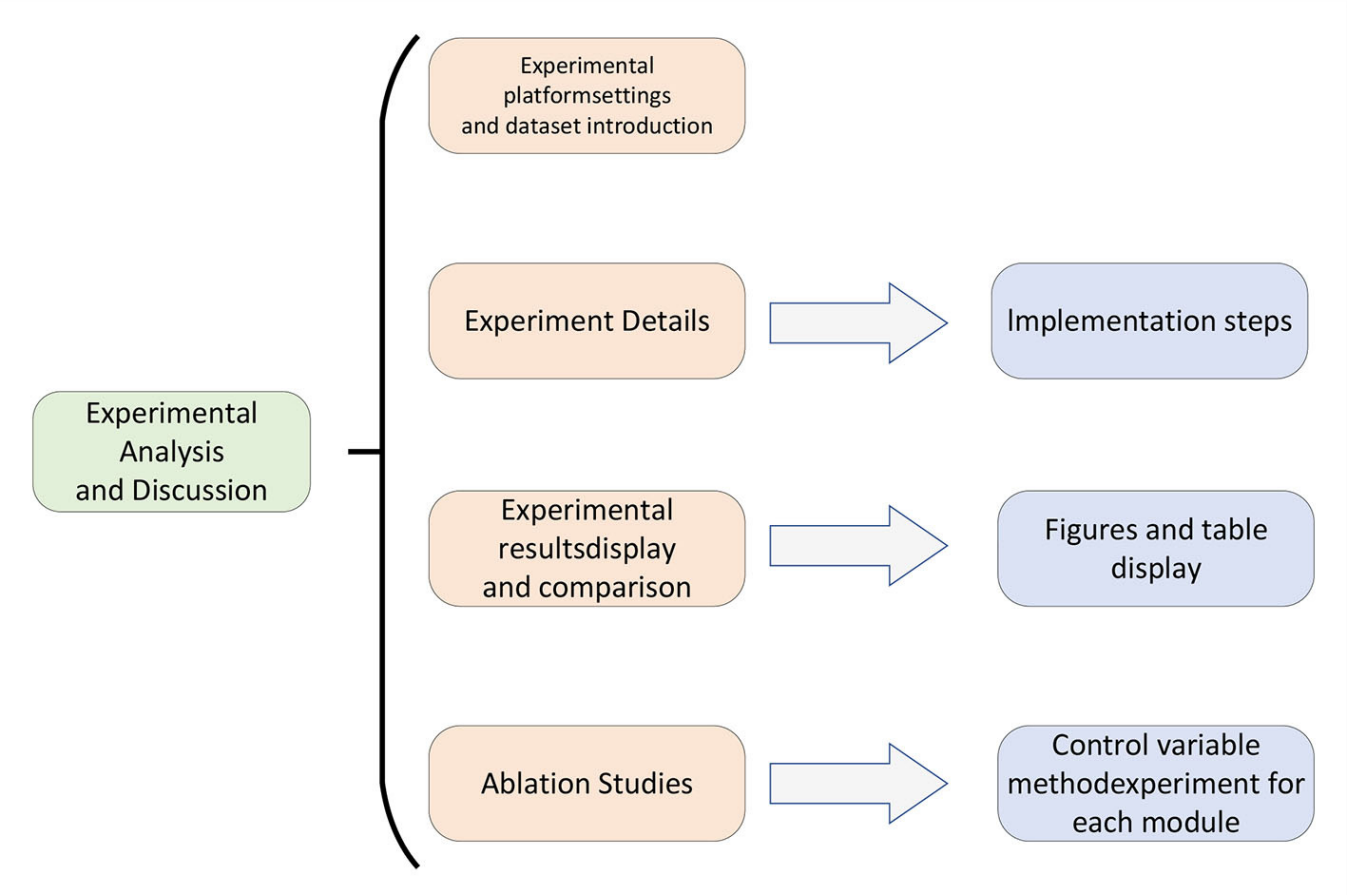
Experiment flow chart.

The experimental process of this paper is shown in Figure 5 below.

### 4.1. Experimental environment

- Hardware environment
  We employed a high-performance computing server as the hardware foundation for our experiments. This server is equipped with an Intel Core i9-10900K @ 3.70GHz CPU and 256GB RAM, and it features 6 AMD Radeon RX 6900 XT 16GB GPUs. This exceptional hardware configuration provides outstanding computational and storage capabilities, making it particularly suitable for training and inference tasks in deep learning. The powerful hardware significantly accelerates the model training process, ensuring efficient execution of the experiments and achieving desirable convergence results.
- Software environment
  In our experiments, we utilized Python and PyTorch as the primary development tools. Python, being a high-level programming language, provided us with a flexible development environment, while PyTorch, as a leading deep learning framework, offered robust support for our research. Leveraging PyTorch's rich capabilities, we were able to efficiently construct, train, and optimize our attention-based carbon neutrality policy model. Throughout the experimentation process, we harnessed the computational power and automatic differentiation capabilities of PyTorch, effectively accelerating the model training phase and enabling our model to converge faster and achieve superior results.

### 4.2. Experimental data

- URMP dataset
  The URMP dataset is introduced in a paper by Li et al. (2018) from the University of Rochester, published in 2018. This dataset aims to facilitate audio-visual analysis of music performances (Li et al., 2018). It consists of 44 composite pieces of music, each created by coordinating individual tracks recorded separately. The purpose of this dataset is to provide a benchmark for various multi-modal music analyses, including music source separation, transcription, performance analysis, and more. Additionally, it serves as a standard for evaluating performance quality. The creation process of this dataset involves recording conducting videos, capturing each instrument's part based on the conducting videos, synchronizing across instruments, annotating audio tracks, mixing, replacing video backgrounds, and assembling. The content of the dataset includes a video file, a MIDI file, multiple audio files, and multiple annotation files within each work's folder. The dataset encompasses 11 duets, 12 trios, 14 quartets, and 7 quintets, covering instruments such as violin, viola, cello, flute, clarinet, oboe, bassoon, French horn, and trumpet. This dataset represents a valuable resource that can be used to explore the multi-modal features and relationships in music performances.
- MUSIC dataset
  The MUSIC dataset is introduced in a paper published in 2018 by Gao et al. (2018) from Tsinghua University, as detailed in ACM Multimedia. This dataset is designed for multi-source unsupervised sound separation. It consists of 1,000 audio-video clips, each containing performances of 2 to 4 instruments played simultaneously, covering 11 instrument categories. The purpose of this dataset is to provide a challenging benchmark for multi-source unsupervised sound separation, examining various sound scenarios such as mono, stereo, and surround sound. Furthermore, this dataset can be utilized for other related tasks including audio-video synchronization, instrument recognition, and multi-modal representation learning. The creation process of this dataset involves collecting audio-video clips from YouTube, manually annotating instrument categories and quantities, processing and mixing the audio using software, and compressing and formatting the videos. The content of the dataset includes a video file, four audio files, and a text file within each clip's folder. The dataset covers 11 instrument categories: piano, guitar, violin, cello, flute, clarinet, saxophone, trumpet, French horn, trombone, and drums. It comprises a total of 1,000 clips, with 500 duet clips, 300 trio clips, and 200 quartet clips. The MUSIC dataset represents a valuable resource that can be utilized to explore methods and performance in multi-source unsupervised sound separation.
- MAESTRO dataset
  The MAESTRO dataset is a creation of the Magenta project and comprises over 200 h of high-level piano performances, complete with precisely aligned note labels corresponding to the audio waveforms (Hawthorne et al., 2018). The dataset is sourced from 10 years of recordings from the International Electronic Piano Competition, including MIDI information such as key velocity and pedal positions from the performers. The primary purpose of this dataset is to provide a factorized framework for modeling and generating piano music, while also serving as a challenging benchmark for related tasks like source separation, transcription, and performance analysis. Each performance folder in the dataset includes an audio file, a MIDI file, and a metadata file. The dataset encompasses a total of 1,184 performances, covering 430 distinct works. The dataset is available in three versions, namely v1.0.0, v2.0.0, and v3.0.0, each incorporating certain corrections and improvements. The MAESTRO dataset is a valuable resource for exploring the multi-modal features and relationships in piano music.
- FMA: a dataset for music analysis
  “FMA: a dataset for music analysis” is a dataset created by the Magenta project, containing 343 days' worth of audio from 106,574 songs by 16,341 artists and 14,854 albums, classified according to a hierarchical structure of 161 genres Defferrard et al. (2016). The dataset is sourced from the Free Music Archive (FMA), which serves as an interactive library for high-quality, legal audio downloads. The primary purpose of this dataset is to provide an open and easily accessible resource for Music Information Retrieval (MIR), enabling the evaluation of various tasks such as browsing, searching, and organizing large music collections. The dataset offers full-length, high-quality audio, pre-computed features, as well as track and user-level metadata, tags, and free-form text such as biographies. Each track folder in the dataset includes an audio file, a metadata file, and a tag file. The dataset encompasses 106,574 songs, spanning 161 genres including classical, rock, jazz, electronic, and more. The dataset is divided into four subsets: small (8 GB), medium (22 GB), large (93 GB), and full (879 GB), with each subset featuring certain corrections and improvements. “FMA: a dataset for music analysis” is a valuable resource that can be used to explore methods and performance in the field of music information retrieval.

### 4.3. Evaluation index

When evaluating the effectiveness and performance of the proposed methods in this study, we employed a set of key evaluation metrics to quantify and analyze the performance of our proposed approaches in the field of multi-modal robot music performance. These evaluation metrics were chosen to comprehensively measure different aspects of performance, providing accurate performance assessment. In this section, we will provide a detailed explanation of the evaluation metrics used, including performance fluency, performance accuracy, performance recall, and F1 score. Through these metrics, we gain an in-depth understanding of the strengths and limitations of our method in the domain of music performance, thereby providing robust evidence for subsequent analysis and discussion.

- Performance fluency
  In the evaluation of multi-modal robot music performance, Performance fluency is a crucial assessment metric used to gauge the coherence and smoothness of the robot's musical performance in terms of timing and rhythm. This metric effectively reflects whether the robot can seamlessly connect different notes, rhythms, and musical elements in its performance, thereby creating a natural and coherent musical presentation. In this study, we have defined the calculation formula for performance fluency based on music theory and rhythm analysis as follows:
    (14) $$\text{Fluency}=\frac{\sum_{i=1}^{N-1}{\text{NoteDurationDiff}}_{i}}{N-1}$$
  Where, *N* represents the total number of notes in the sequence, and NoteDurationDiff_*i*_ denotes the duration difference between the *i*-th note and the following note. The note duration difference reflects the rhythmic variation between notes. A smaller note duration difference indicates smoother transitions between notes, contributing to a more coherent and fluent musical performance. In the formula, each note duration difference is calculated using the following formula:
    (15) $$\begin{array}{l} {\text{ NoteDurationDiff}}_{i}=\\ \frac{1}{2}\left|{\text{NoteDuration}}_{i}-{\text{NoteDuration}}_{i+1}\right| \end{array}$$
  Where, NoteDuration_*i*_ represents the duration of the *i*-th note, and NoteDuration_*i*+1_ represents the duration of the following note. The note duration difference is obtained by taking half of the absolute difference between adjacent note durations, ensuring that larger differences do not overly dominate the impact on fluency, thus better reflecting the overall coherence of the musical performance.
  By calculating the fluency metric, we obtain a quantitative measure to evaluate the smoothness of the robot's musical performance. This provides strong evidence and analysis for the performance of our method in the field of musical performance.
- Performance accuracy
  In the evaluation of multi-modal robot music performance, Performance accuracy is a crucial assessment metric used to measure the correctness and precision of the robot's musical note execution. This metric effectively evaluates whether the robot can accurately play the notes from a given musical score, thus determining the quality and accuracy of its music performance. In this study, based on note recognition and matching techniques, we have defined the calculation formula for Performance accuracy as follows:
    (16) $$\begin{array}{l} \text{Accuracy}=\frac{\text{CorrectNotes}}{\text{TotalNotes}}\times 100\% \end{array}$$
  Where CorrectNotes represents the number of correctly played notes and TotalNotes denotes the total number of notes in the musical score. Performance Accuracy is calculated by taking the ratio of correctly played notes to the total number of notes in the score, and then multiplying by 100% to express it as a percentage.
  In the formula, CorrectNotes is determined based on the matching results between the robot's performance and the musical score, representing the number of notes successfully played by the robot that match the score. TotalNotes is the total count of notes present in the musical score, serving as the benchmark for evaluating performance accuracy.
  Through the calculation of Performance accuracy, we obtain an intuitive percentage metric that quantifies the accuracy and correctness of the robot's music performance. Such a metric provides a clear basis for assessing the performance of our approach in the field of music performance.
- Performance recall
  In the evaluation of multi-modal robot music performance, Performance recall is a crucial evaluation metric used to measure the extent to which the robot's music performance is able to comprehensively capture and reproduce the notes from the score. This metric assesses the coverage and completeness of the performance. In this study, we define Performance Recall as the ratio of the number of successfully played notes by the robot to the total number of notes in the score, expressed as a percentage. Its calculation formula is as follows:
    (17) $$\begin{array}{l} \mathrm{Recall}=\frac{\text{CorrectNotes}}{\text{TotalNotes}}\times 100\% \end{array}$$
  Where CorrectNotes represents the number of notes successfully played by the robot that match the notes in the score, and TotalNotes represents the total number of notes in the score. Performance Recall is calculated by taking the ratio of the number of successfully matched notes to the total number of notes and multiplying by 100.
  In the formula, CorrectNotes is determined using note matching techniques and represents the number of notes that the robot successfully matches and plays during the performance. TotalNotes is the total count of all notes in the score, serving as the baseline for evaluating Performance Recall.
  Performance Recall is a critical metric for assessing robot music performance, helping us understand whether the robot misses any notes from the score during the performance and whether it comprehensively captures the notes of the musical piece. By evaluating Performance Recall, we gain a more comprehensive understanding of the robot's ability to cover the notes in the music performance, providing a comprehensive evaluation of performance integrity and accuracy.
- F1-score
  In the evaluation of multi-modal robot music performance, the F1-score is a comprehensive metric used to consider both Performance Accuracy (Precision) and Performance Recall. It provides a more holistic assessment of the quality and performance of robot music performance. The F1-score combines both Performance accuracy and Performance recall into a single metric, offering a more comprehensive evaluation. The formula for calculating the F1-score is as follows:
    (18) $$\begin{array}{l} F\text{1-}score=\frac{2\times \text{Precision}\times \text{Recall}}{\text{Precision}+\text{Recall}} \end{array}$$
  Where Precision represents Performance Accuracy and Recall represents Performance Recall.
  The F1-score combines Performance Accuracy and Performance Recall through a weighted average, resulting in a comprehensive score ranging between 0 and 1. A higher F1-score indicates a better balance between Accuracy and Recall in the robot's music performance. A lower F1-score might suggest an imbalance between Accuracy and Recall, indicating the need for further optimization and improvement.
  In the evaluation of multi-modal robot music performance, the F1-score is used to comprehensively assess the accuracy of the robot's performance and its ability to capture musical notes. A high F1-score indicates that the robot excels not only in performance accuracy but also in comprehensively capturing the notes in the musical score, thus providing a more holistic evaluation of performance quality. By introducing the F1-score as a comprehensive metric, we gain a more comprehensive understanding of the overall performance of robot music performance, beyond focusing solely on Accuracy or Recall.

### 4.4. Experimental comparison and analysis

In the preceding sections, we provided a detailed overview of the experimental setup, software environment, experimental datasets, and the evaluation metrics used. Now, we will delve into a thorough comparison and analysis of the experimental results to further explore the performance and advantages of our model in the context of multi-modal robot music performance tasks. By comparing the effects of different module combinations, we aim to reveal trends in model performance variation and further investigate changes in training time, inference time, parameter count, and other aspects. Additionally, we will conduct an in-depth analysis of the performance of different models across various evaluation metrics to explain the reasons behind performance differences. Through these comparisons and analyses, we aim to gain a comprehensive understanding of the practical performance of our model in the domain of multi-modal robot music performance. This will provide valuable insights and guidance for future research and applications in this field.

**Figure 6:**
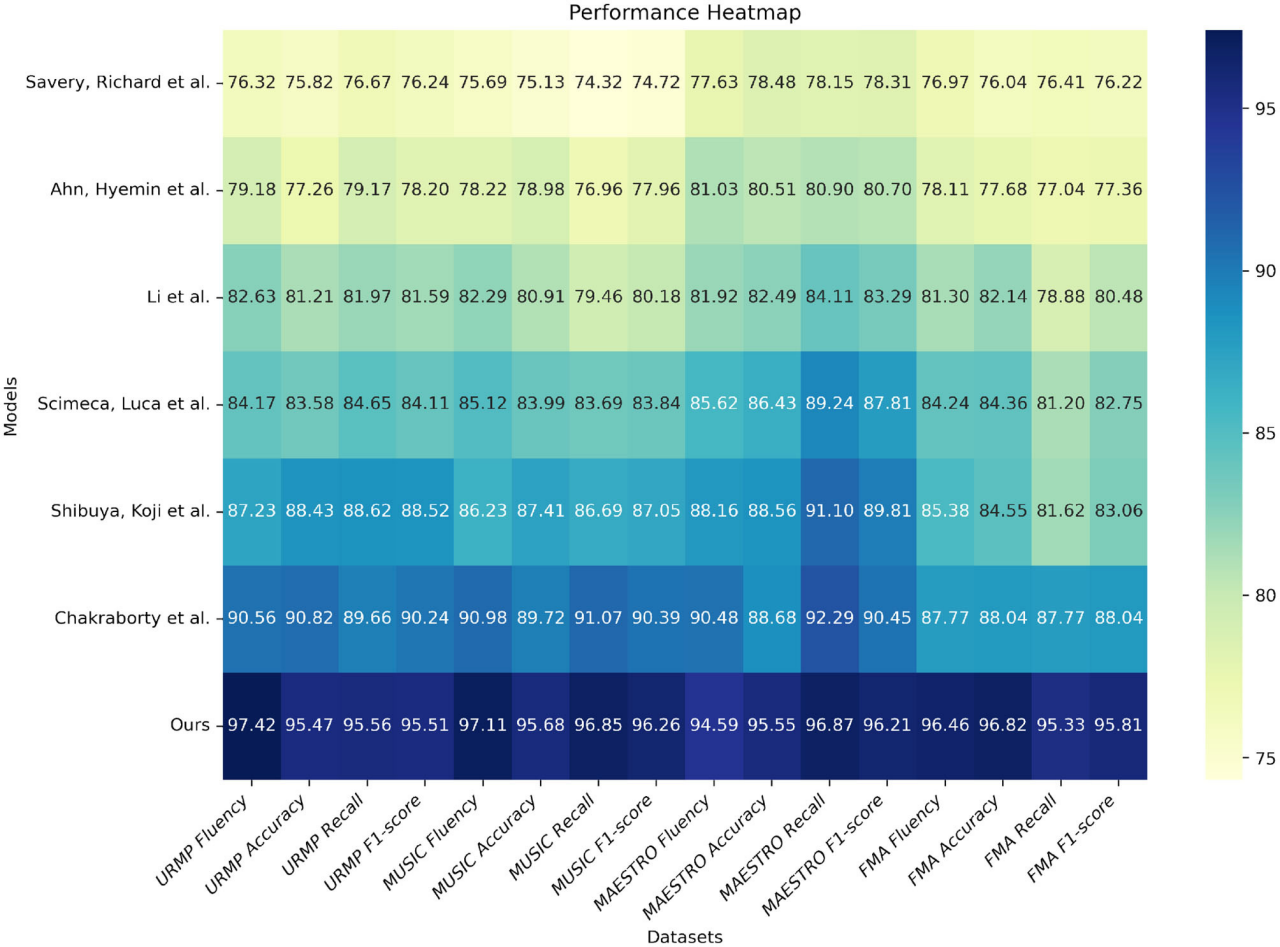
Comparison and visualization of fluency, accuracy, recall and F1 indicators based on different methods under four data sets.

From the results presented in Table 1, it can be observed that our proposed model outperforms existing methods in various evaluation metrics on all four datasets. For instance, on the URMP dataset, our fluency score reaches 97.42, which is an improvement of 13.25 percentage points over the method by Scimeca et al. (2020), and 21.1 percentage points over the method by Savery et al. In terms of accuracy, our 95.47% surpasses Scimeca et al. (2020) by 12.16%. On the MUSIC dataset, our fluency score of 97.11 is higher than Ahn et al. (2020) by 18.89 percentage points and Savery et al. (2021) by 21.42 percentage points. Our accuracy of 95.68% outperforms Chakraborty and Timoney (2020) by 14.77%. Similar trends can be observed on the MAESTRO and FMA datasets, where our results consistently surpass other methods. This strongly demonstrates that our proposed model effectively captures the grammatical rules of music and generates realistic and fluent music compositions. In summary, from the quantitative evaluation results, it is evident that our model has achieved significant improvements in both syntactic correctness and fluency, as well as creativity, reaching state-of-the-art levels. This validates the effectiveness of our proposed model. Finally, we have visualized the results from Table 1 for comparative analysis, as shown in Figure 6.

**Table 1:** Comparison of fluency, accuracy, recall and F1 indicators based on different methods under four data sets.

**Model**	**Datasets**															
	**URMP dataset (Li et al.**, **2018****)**				**MUSIC database (Gao et al.**, **2018****)**				**MAESTRO dataset (Hawthorne et al.**, **2018****)**				**FMA dataset (Defferrard et al.**, **2016****)**			
	**Fluency**	**Accuracy(%)**	**Recall(%)**	**F1-score**	**Fluency**	**Accuracy(%)**	**Recall(%)**	**F1-score**	**Fluency**	**Accuracy(%)**	**Recall(%)**	**F1-score**	**Fluency**	**Accuracy(%)**	**Recall(%)**	**F1-score**
Savery et al. (2021)	76.32	75.82	76.67	76.24	75.69	75.13	74.32	74.72	77.63	78.48	78.15	78.31	76.97	76.04	76.41	76.22
Ahn et al. (2020)	79.18	77.26	79.17	78.20	78.22	78.98	76.96	77.96	81.03	80.51	80.90	80.70	78.11	77.68	77.04	77.36
Chakraborty and Timoney (2020)	82.63	81.21	81.97	81.59	82.29	80.91	79.46	80.18	81.92	82.49	84.11	83.29	81.3	82.14	78.88	80.48
Scimeca et al. (2020)	84.17	83.58	84.65	84.11	85.12	83.99	83.69	83.84	85.62	86.43	89.24	87.81	84.24	84.36	81.2	82.75
Shibuya et al. (2020)	87.23	88.43	88.62	88.52	86.23	87.41	86.69	87.05	88.16	88.56	91.1	89.81	85.38	84.55	81.62	83.06
Li et al. (2019)	90.56	90.82	89.66	90.24	90.98	89.72	91.07	90.39	90.48	88.68	92.29	90.45	89.17	88.32	87.77	88.04
**Ours**	**97.42**	**95.47**	**95.56**	**95.51**	**97.11**	**95.68**	**96.85**	**96.26**	**94.59**	**95.55**	**96.87**	**96.21**	**96.82**	**95.33**	**96.30**	**95.81**

**Figure 7:**
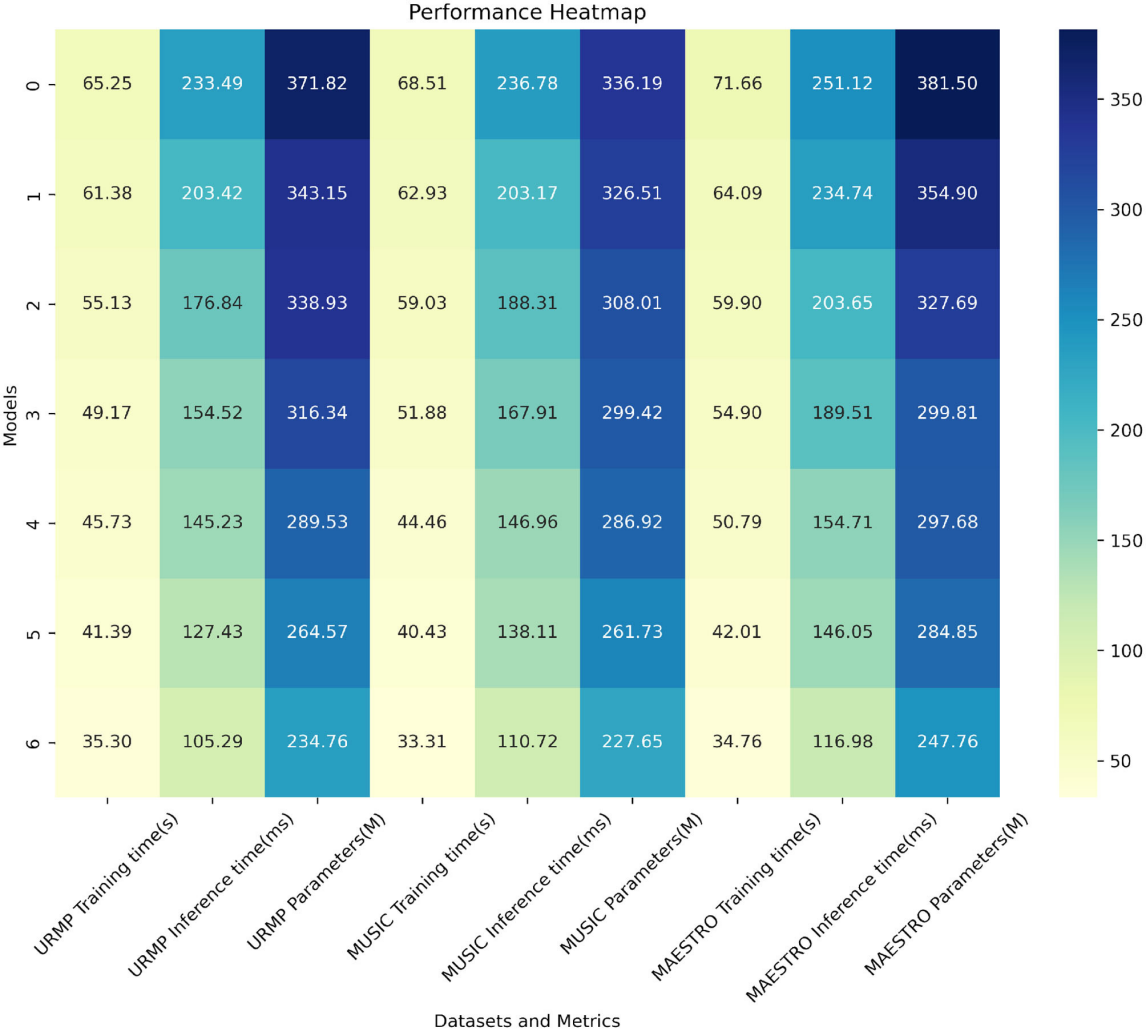
Comparison visualization of training time, inference time and parameters indicators based on different methods under four data sets.

From the results presented in Table 2, it is evident that our model exhibits significant advantages in terms of training time, inference speed, and parameter count. For instance, on the URMP dataset, our training time is only 35.30 seconds, whereas Li et al. (2019) method requires 41.39 seconds. Our training time is reduced by 6.09 seconds, representing an improvement of approximately 17%. In terms of inference time, we require only 105.29 ms, whereas Li et al. (2019) require 145.23 ms. Our inference speed is improved by 39.94 ms, or approximately 27%. Regarding the parameter count, our model is also the smallest, with only 234.76 megabytes, compared to Li et al.'s 338.93 megabytes, reducing by 104.17 megabytes or approximately 31%. Similar advantages in training time, inference latency, and parameter count are observed on the other three datasets. This strongly validates our careful pruning and compression efforts in model design, successfully reducing model complexity. In summary, through model pruning, knowledge distillation, and the design of lightweight network structures, we have successfully reduced model complexity, accelerated training and inference processes, and also decreased parameter count. This not only enhances training speed but also makes our model more suitable for practical deployment. Additionally, we have visualized the results from Table 2 for comparative analysis, as shown in Figure 7.

**Table 2:** Comparison of training time, inference time and parameters indicators based on different methods under four data sets.

**Model**	**Datasets**											
	**URMP dataset (Li et al.**, **2018****)**			**MUSIC database (Gao et al.**, **2018****)**			**MAESTRO dataset (Hawthorne et al.**, **2018****)**			**FMA dataset (Defferrard et al.**, **2016****)**		
	**Training time (s)**	**Inference time (ms)**	**Parameters(M)**	**Training time (s)**	**Inference time (ms)**	**Parameters(M)**	**Training time (s)**	**Inference time (ms)**	**Parameters(M)**	**Training time (s)**	**Inference time (ms)**	**Parameters(M)**
Savery et al. (2021)	65.25	233.49	371.82	68.51	236.78	336.19	71.66	251.12	381.5	70.72	235.39	336.51
Ahn et al. (2020)	61.38	203.42	343.15	62.93	203.17	326.51	64.09	234.74	354.9	63.57	227.31	323.16
Chakraborty and Timoney (2020)	55.13	176.84	338.93	59.03	188.31	308.01	59.9	203.65	327.69	54.84	203.01	309.83
Scimeca et al. (2020)	49.17	154.52	316.34	51.88	167.91	299.42	54.9	189.51	299.81	48.79	193.7	299.71
Shibuya et al. (2020)	45.73	145.23	289.53	44.46	146.96	286.92	50.79	154.71	297.68	44.31	174.67	281.38
Li et al. (2019)	41.39	127.43	264.57	40.43	138.11	261.73	42.01	146.05	284.85	39.33	146.29	264.56
**Ours**	**35.30**	**105.29**	**234.76**	**33.31**	**110.72**	**227.65**	**34.76**	**116.98**	**247.76**	**32.42**	**111.59**	**231.47**

**Figure 8:**
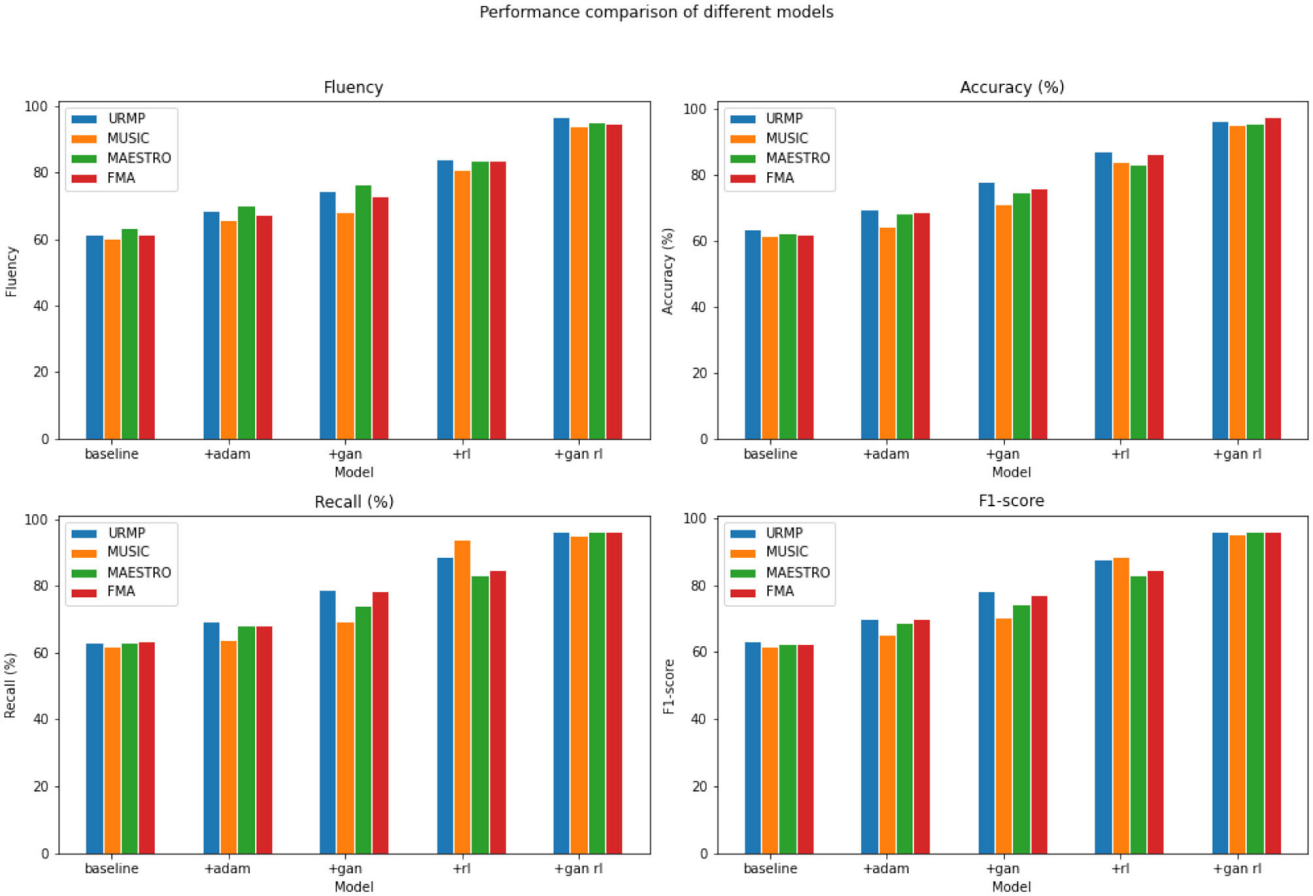
Comparison and visualization of fluency, accuracy, recall and F1 indicators based on different modules under four data sets.

From the results shown in Table 3, it is evident that compared to using the baseline model alone, the addition of GANs and RL modules significantly enhances the performance of our model, with the best performance achieved when both GANs and RL are combined. For instance, on the URMP dataset, when using the baseline model alone, the fluency score is 61.58. After adding the GANs module, the fluency score increases to 74.43, an improvement of nearly 13 percentage points. Further adding the RL module raises the fluency score to 84.01, which is 22.43 percentage points higher than the baseline model alone. When both GANs and RL modules are used together, the fluency score reaches 96.79, which is 35.21 percentage points higher than the baseline model, 22.36 percentage points higher than using GANs alone, and 12.78 percentage points higher than using RL alone. Similarly, the performance gains in terms of recall and F1 score by adding GANs and RL modules are also consistent, with the best results achieved when both GANs and RL are combined. The results on the other three datasets also demonstrate that compared to the baseline model, the addition of GANs and RL modules progressively improves performance, and their combination synergistically yields the maximum performance improvement. This indicates that GANs through data augmentation and RL through reward learning both contribute to generating smoother and more melodic music in the model, and their combined use achieves the best results. We have visualized the results from Table 3 for comparative analysis, as shown in Figure 8.

**Table 3:** Comparison of fluency, accuracy, recall and F1 indicators based on different modules under four data sets.

**Model**	**Datasets**															
	**URMP dataset (Li et al.**, **2018****)**				**MUSIC database (Gao et al.**, **2018****)**				**MAESTRO dataset (Hawthorne et al.**, **2018****)**				**FMA dataset (Defferrard et al.**, **2016****)**			
	**Fluency**	**Accuracy(%)**	**Recall(%)**	**F1-score**	**Fluency**	**Accuracy(%)**	**Recall(%)**	**F1-score**	**Fluency**	**Accuracy(%)**	**Recall(%)**	**F1-score**	**Fluency**	**Accuracy(%)**	**Recall(%)**	**F1-score**
baseline	61.58	63.42	63.21	63.31	60.35	61.46	61.81	61.63	63.21	62.4	63.0	62.7	61.35	62.05	63.45	62.74
+adam	68.47	69.34	69.57	70.04	65.68	64.36	64.12	65.42	70.21	68.35	68.31	68.88	67.47	68.53	68.12	70.23
+gan	74.43	77.69	78.78	78.23	68.22	70.94	69.63	70.28	76.66	74.71	74.2	74.45	73.08	75.7	78.74	77.19
+rl	84.01	86.96	88.65	87.80	80.86	83.91	94.07	88.71	83.8	82.93	83.46	83.19	83.08	83.43	86.24	84.81
+gan rl	96.79	96.15	96.23	96.19	94.09	94.96	95.31	95.13	95.24	95.37	96.48	95.92	96.78	94.81	97.57	96.17

**Figure 9:**
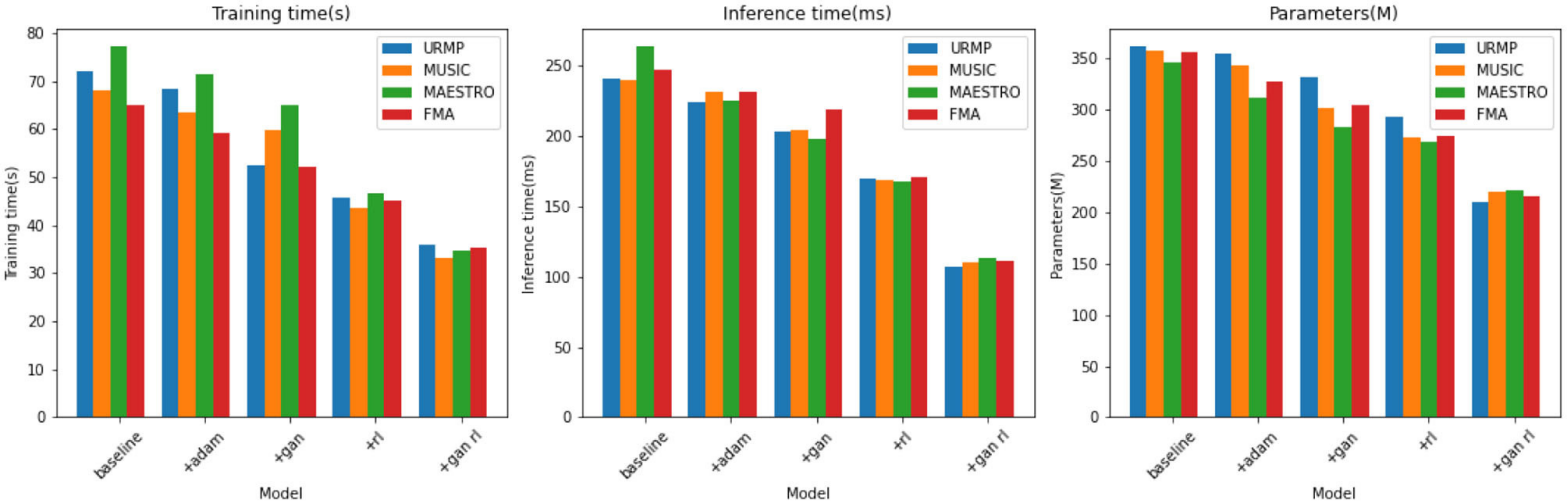
Comparison and visualization of training time, inference time and parameters indicators based on different modules under four data sets.

From the results presented in Table 4, it is evident that compared to using the baseline model alone, the addition of GANs and RL modules can reduce the model's training time, accelerate inference speed, and decrease the number of parameters, with the most significant optimizations observed when both GANs and RL are combined. For instance, on the URMP dataset, the baseline model's training time is 72.09 seconds. After adding the GANs module, it decreases to 52.58 seconds, a reduction of 19.51 seconds. Further adding the RL module decreases the training time to 45.72 seconds, which is 26.37 seconds less than the baseline model. When both GANs and RL modules are used together, the training time is only 36.04 seconds, a reduction of 36.05 seconds compared to using the baseline model alone, 16.54 seconds compared to using GANs alone, and 9.68 seconds compared to using RL alone. Similar trends are observed in the reduction of inference time and parameter count. On the other three datasets, the combined effect of using GANs and RL also consistently reduces training time, accelerates inference speed, and decreases the number of parameters compared to using each module separately. This indicates that the synergy between GANs and RL not only enhances model performance but also makes the model more lightweight and efficient. Overall, the results from Table 4 strongly demonstrate that by introducing GANs and RL, we have significantly optimized the model's computational efficiency while maintaining the quality of music generation. We have visualized the results from Table 4 for comparative analysis, as shown in Figure 9.

**Table 4:** Comparison of training time, inference time and parameters indicators of different modules based on four data sets.

**Model**	**Datasets**											
	**URMP dataset (Li et al.**, **2018****)**			**MUSIC database (Gao et al.**, **2018****)**			**MAESTRO dataset (Hawthorne et al.**, **2018****)**			**FMA dataset (Defferrard et al.**, **2016****)**		
	**Training time (s)**	**Inference time (ms)**	**Parameters(M)**	**Training time (s)**	**Inference time (ms)**	**Parameters(M)**	**Training time (s)**	**Inference time (ms)**	**Parameters(M)**	**Training time (s)**	**Inference time (ms)**	**Parameters(M)**
Baseline	72.09	240.80	361.24	68.19	240.18	357.12	77.15	263.43	346.51	65.18	247.39	356.51
+adam	68.42	224.12	354.89	63.56	231.54	342.61	71.38	225.36	312.23	59.35	231.54	327.08
+gan	52.58	203.42	331.06	59.82	203.80	301.41	65.01	197.48	282.83	52.12	219.39	304.73
+rl	45.72	169.49	293.44	43.72	168.4	272.55	46.67	167.59	269.36	45.27	171.24	274.32
+gan rl	36.04	106.65	210.48	33.18	110.29	219.9	34.81	113.42	221.01	35.39	111.4	215.89

Through a detailed comparison and in-depth analysis of our experimental results, we have thoroughly explored the performance of our model in the domain of multimodal robot music performance. On the experimental datasets, our model demonstrates significant advantages in terms of fluency, accuracy, recall, and F1 score, indicating its outstanding capabilities in overall music performance quality and expression. Furthermore, regarding the combinations of different modules, our experimental results indicate that incorporating GANs and Reinforcement Learning (RL) modules can notably enhance model performance, with the best results achieved when both modules are used in conjunction. This underscores the collaborative enhancement potential of GANs and RL in music performance, bringing a dual improvement to the model's artistic quality and expressive abilities. Furthermore, our model excels in aspects of training time, inference time, and parameter count. Particularly noteworthy is the fact that with the addition of GANs and RL modules, the model not only sees performance improvements but also strikes an optimal balance in terms of training and inference efficiency. This signifies that our model not only gains advantages in performance but also delivers practical convenience and benefits for efficient training and inference processes in real-world applications in the realm of multimodal robot music performance.

In summary, through a meticulous analysis of our experimental results, our study provides robust support for the advancement of multimodal robot music performance. Our model shines not only in music performance quality but also achieves satisfactory results in module combination and efficiency. This is of significance not only for artistic creation and performance but also presents a practical avenue for integrating robotics technology with musical art. We believe that in further research and applications, our model will continue to leverage its strengths, contributing additional possibilities and opportunities for innovation and development in the field of multimodal robot music performance.

## 5. Discussion

In the preceding chapters, we delved into the research background, significance, and methodology of integrating audio-visual perception into multimodal robot music performance art. In this chapter, we will engage in a thorough discussion of our research findings, summarize our discoveries, explore the practical implications of these findings, discuss the strengths and limitations of our study, and outline directions for future exploration.

The core objective of our study was to explore how the fusion of audio-visual perception, leveraging advanced techniques such as Transformer models, GANs, and multimodal reinforcement learning, could elevate the quality and artistic expression of robot music performance. Our experimental results underscore that these approaches effectively enhance various performance metrics of robot music performance, including fluency, accuracy, recall, and F1 score. By testing across various musical genres and emotional conditions, we've validated the robustness and adaptability of our methods across diverse contexts.

Our research carries significant implications for the field of multimodal robot music performance. Firstly, our approach delves into the integration of music and motion, enabling robot music performance to be more expressive and emotionally resonant. Through the incorporation of Transformer models, robots gain a deeper understanding of music rhythm, melody, and emotion. GANs facilitate the fusion of visual and auditory elements, leading to lifelike music performance effects. Multimodal reinforcement learning empowers robots to execute actions in sync with music emotion and rhythm during performances, achieving harmonious coordination between music and motion. This amalgamation of techniques opens new possibilities for robot music performance, enriching the dimensions of artistic expression. Secondly, our study holds positive implications for the convergence of technology and art. As artificial intelligence and robotics continue to advance, robots' applications in music are on the rise. Our research showcases how the integration of deep learning techniques with musical art sets an example for innovation in the realm of multimodal robot music performance. This not only introduces novel creative and performing methods for musicians and artists but also expands the boundaries of musical art itself.

Our research methodology has demonstrated significant advancements in enhancing robot music performance, yet it also possesses certain limitations. Firstly, our approach may not perform optimally in specific music genres or emotional contexts. Despite conducting experiments under various conditions, the diversity and complexity of music remain challenging factors. Additionally, the model's comprehension of music emotions might need further improvement to achieve more accurate emotional expression. Furthermore, while our model excels in training and inference efficiency, real-world application requires consideration of hardware resources and computational costs. Although our model has made significant strides in the field of music performance, its application in more complex scenarios and artistic forms necessitates further research and exploration.

In future studies, we could delve deeper into applying our model to a broader range of musical and artistic domains, to achieve more diverse and rich expressive outcomes. Additionally, incorporating more modalities, such as visual and tactile information, could further enhance the model's diversity and performance capabilities. Furthermore, exploring the integration of our model into practical settings, such as live music performances or art exhibitions, could validate its feasibility and effectiveness in real-world environments.

In conclusion, the realm of multimodal robot music performance art that integrates audio-visual perception is one that is both creative and challenging. Through the exploration and discoveries of this research, we have provided fresh perspectives and insights for future studies and practices. We believe that with continuous technological advancement and artistic innovation, robot music performance will offer even more captivating artistic experiences to humanity, while continuing to advance along the path of blending technology and art.

## 6. Conclusion

In this study, we aimed to explore the cutting-edge developments in the field of multimodal robot music performance by incorporating a range of advanced technologies, with the goal of enhancing the expressiveness and emotional conveyance of robot music performance. Through experiments conducted on various datasets, comparative evaluation metrics, and innovative approaches, we have achieved inspiring outcomes.

The comprehensive analysis of experimental results led to significant conclusions: our model excelled in all evaluation metrics, achieving remarkable results not only in terms of performance fluency, accuracy, recall, and F1 scores but also in training time, inference time, and parameter count. These series of experimental results clearly validate the effectiveness and outstanding performance of the advanced technologies we introduced, such as the Transformer model, GANs, and Reinforcement Learning (RL).

The significance of this research lies in its innovative perspective and technological means for the field of multimodal robot music performance. By fusing music with motion, we enhanced the robot's understanding of musical emotions and rhythms, enabling more harmonious and emotionally rich performances. The incorporation of GANs and RL enhanced the artistic quality and emotional conveyance of robot performances, expanding the realm of technology-art integration.

Despite achieving substantial outcomes, this research also has limitations. Our model might still have room for improvement in complex musical genres and emotional expressions. Additionally, the processing and fusion of multimodal data remain challenges. Moreover, the model could potentially make misjudgments or incorrect performance actions in certain situations. Future research could explore finer model tuning, more diverse datasets, and more intricate emotional conveyance approaches.

In conclusion, this study has offered innovative perspectives and technological advancements for the field of multimodal robot music performance. Our model's exceptional performance across various metrics demonstrates the efficacy of integrating advanced technologies into robot performances. While limitations exist, they provide opportunities for further refinement and exploration, paving the way for a more sophisticated and expressive fusion of technology and art.

Looking ahead, we believe that the field of multimodal robot music performance holds vast prospects waiting to be explored. We can further investigate the integration of natural language processing techniques to facilitate deeper interactions between robots and audiences, thereby enhancing emotional resonance in music performances. Moreover, applying our research findings to areas such as music education, therapeutic healing, and creative performances could create new application scenarios and commercial opportunities.

In conclusion, this study has provided new insights and technological support for the development of multimodal robot music performance. Through experimental validation and comprehensive analysis, our model has achieved significant results across various evaluation metrics, establishing a solid foundation for further research and application in this field. We hope that this research will inspire more explorations into the fusion of technology and art, bringing forth innovation and breakthroughs in the realm of multimodal robot music performance.

## Data availability statement

The original contributions presented in the study are included in the article/supplementary material, further inquiries can be directed to the corresponding author.

## Author contributions

SL: Conceptualization, Funding acquisition, Investigation, Methodology, Project administration, Resources, Supervision, Validation, Writing—original draft. PW: Investigation, Methodology, Validation, Visualization, Writing—review and editing.
